# Towards an Improved Pathological Node Classification for Prognostic Stratification of Patients With Oral Cavity Squamous Cell Carcinoma: Results From a Nationwide Registry Study

**DOI:** 10.3389/fonc.2022.910158

**Published:** 2022-06-28

**Authors:** Chung-Jan Kang, Yu-Wen Wen, Shu-Ru Lee, Shu-Hang Ng, Chi-Ying Tsai, Li-Yu Lee, Ying-Hsia Chu, Chien-Yu Lin, Kang-Hsing Fan, Hung-Ming Wang, Chia-Hsun Hsieh, Chih-Hua Yeh, Chih-Hung Lin, Chung-Kan Tsao, Tuan-Jen Fang, Shiang-Fu Huang, Li-Ang Lee, Ku-Hao Fang, Yu-Chien Wang, Wan-Ni Lin, Li-Jen Hsin, Tzu-Chen Yen, Nai-Ming Cheng, Chun-Ta Liao

**Affiliations:** ^1^ Department of Otorhinolaryngology, Head and Neck Surgery, Chang Gung Memorial Hospital and Chang Gung University, Taoyuan, Taiwan; ^2^ Clinical Informatics and Medical Statistics Research Center, Chang Gung University, Taoyuan, Taiwan; ^3^ Division of Thoracic Surgery, Chang Gung Memorial Hospital, Taoyuan, Taiwan; ^4^ Research Service Center for Health Information, Chang Gung University, Taoyuan, Taiwan; ^5^ Department of Diagnostic Radiology, Chang Gung Memorial Hospital and Chang Gung University, Taoyuan, Taiwan; ^6^ Department of Oral and Maxillofacial Surgery, Chang Gung Memorial Hospital, Chang Gung University, Taoyuan, Taiwan; ^7^ Department of Pathology, Chang Gung Memorial Hospital and Chang Gung University, Taoyuan, Taiwan; ^8^ Department of Radiation Oncology, Chang Gung Memorial Hospital and Chang Gung University, Taoyuan, Taiwan; ^9^ Department of Medical Oncology, Chang Gung Memorial Hospital and Chang Gung University, Taoyuan, Taiwan; ^10^ Department of Plastic and Reconstructive Surgery, Chang Gung Memorial Hospital and Chang Gung University, Taoyuan, Taiwan; ^11^ Department of Nuclear Medicine and Molecular Imaging Center, Chang Gung Memorial Hospital and Chang Gung University, Taoyuan, Taiwan

**Keywords:** oral cavity squamous cell carcinoma, positive lymph nodes, lymph node ratio, log odds of positive lymph nodes, extra-nodal extension, cancer registry, survival outcomes

## Abstract

**Background:**

To assess the prognostic significance of different nodal parameters [i.e., number of pathologically positive nodes, log odds of positive lymph nodes, lymph node ratio (LNR), and extra-nodal extension (ENE)] in Taiwanese patients with oral cavity squamous cell carcinoma (OCSCC), and to devise an optimized pN classification system for predicting survival in OCSCC.

**Methods:**

A total of 4287 Taiwanese patients with first primary OCSCC and nodal metastases were enrolled. Cox proportional hazards regression analysis with the spline method was applied to identify the optimal cut-off values for LNR, log odds of positive lymph nodes, and number of pathologically positive nodes.

**Results:**

On multivariable analysis, we identified a LNR ≥0.078/0.079, the presence of at least three pathologically positive nodes, and ENE as independent prognosticators for 5-year disease-specific survival (DSS) and overall survival (OS) rates. We therefore devised a four-point prognostic scoring system according to the presence or absence of each variable. The 5-year DSS and OS rates of patients with scores of 0−3 were 70%/62%/50%/36% (*p <*0.0001) and 61%/52%/40%25%, respectively (*p <*0.0001). On analyzing the AJCC 2017 pN classification, patients with pN3a displayed better survival rates than those with pN2 disease. The 5-year DSS and OS rates of patients with pN1/pN2/pN3a/pN3b disease were 72%/60%/67%/43% (*p <*0.0001) and 63%/51%/67%/33%, respectively (*p <*0.0001).

**Conclusions:**

Three nodal parameters (i.e., a LNR ≥0.078/0.079, the presence of at least three pathologically positive nodes, and ENE) assessed in combination provided a better prognostic stratification than the traditional AJCC pN classification.

## Introduction

Conventional treatment of first primary oral cavity squamous cell carcinoma (OCSCC) is based on surgical resection. Furthermore, the use of adjuvant therapy has become standard clinical practice when postoperative clinicopathological risk factors (RFs) are present (1). The importance of wide tumor excision margins and thorough neck dissections to achieve favorable outcomes is well established (2), and the presence of pathological node metastases (pN+) has been shown to be independently related to a poor prognosis (3). In this scenario, a more precise pN classification has the potential to offer a more patient-tailored approach through an improved prognostic stratification.

Compared with the 2010 edition, the American Joint Committee on Cancer (AJCC) 2017 Staging Manual has introduced a novel nodal classification termed pN3b – which includes pN2−3 disease (according to the 2010 edition) and extra-nodal extension (ENE) (4). While the presence of pN3b disease in patients with resected OCSCC generally portends a poor prognosis, the survival outcomes within this subgroup remain heterogeneous (5). In the era of the AJCC 2010 staging criteria (i.e., before the introduction of the pN3b classification), there have been attempts to improve the pN classification by taking into account the number of dissected and/or pathologically positive nodes (6–36). The number of involved nodes and the lymph node ratio (LNR, calculated as the number of positive nodes divided by the number of dissected nodes) have been shown to independently predict survival outcomes (12–36). Other studies have also concluded that the log odds of positive lymph nodes can serve as a reliable prognostic variable (6–11).

Following the introduction of the pN3b classification in the AJCC 2017 Staging Manual, only few studies have assessed the prognostic value of nodal parameters (5, 37–39). In addition, novel lymph node-related variables have been rarely examined along with the traditional AJCC pN classification (6, 7, 37). However, the issue whether nodal parameters would outperform the AJCC 2017 pN classification system in terms of prognostic stratification is unresolved. Therefore, the purpose of this nationwide registry-based cohort study was to assess the prognostic significance of different node-related variables (i.e., number of pathologically positive nodes, log odds of positive lymph nodes, LNR, and ENE) in Taiwanese patients with OCSCC. We also devised an optimized pN classification system for the prediction of survival outcomes.

## Materials and Methods

### Study Setting

Data for this study were mainly obtained from the Taiwanese Cancer Registry Database (TCRD) “long-form” – which includes the large majority (>98%) of patients with OCSCC referred to the largest Taiwanese hospitals. In general, the TCRD complies with the principles outlined by the American College of Surgeons “Standards for Oncology Registry Entry (STORE)”, including information on histology grade (40). The most recent version of the TCRD has prospectively recorded information on cancer stage, tumor relapses, and treatment modalities which were unavailable in the previous release (termed “short-form”). Starting from 2011, data on ENE, margin status, and depth of invasion (DOI) were also collected. The secondary data source was the Taiwanese National Health Insurance Research Dataset (TNHIRD). The registry can be openly accessed from university hospitals (Health and Welfare Data Science Center) in Taiwan through the Taiwanese Ministry of Health and Welfare. The study was approved by the Chung Gung Memorial Hospital Institutional Review Board (IRB; approval number: 201801398B0A3). All procedures complied with the principles of the Helsinki Declaration. The need for written informed consent was waived due to the study design.

### Treatment Protocol and Follow-Up Protocol

As part of its continued effort to improve the quality of cancer care, the Taiwan Health Promotion Administration has taken initiative to promote multidisciplinary team care (MDTC) and multidisciplinary case management as of April 2003. Because outcomes in patients with OCSCC are largely dependent on the type of surgical approach and the use of adjuvant therapy, a comprehensive strategy for decision-making, therapy, clinical management, and follow-up is mandatory in areas where betel quid chewing is endemic. Starting from these premises, all of the Taiwanese hospital specialized in treating OCSCC began implementing an MDTC approach as of January 2004. In general, the follow-up protocols were in accordance with the NCCN treatment guidelines (41).

### Data Collection

Until 2011, patients were staged according to the AJCC Staging Manual (seventh edition) using data from the TCRD “long form”. We subsequently applied the AJCC 2017-2018 Staging Manual (eight edition) based on the most recent version of the dataset that included information on both ENE and DOI. Data were analyzed in November 2021 by taking into account the most recent TCRD (2017 release) and TNHIRD (2019 release) data sets. Disease-specific survival (DSS) and overall survival (OS) were calculated using events recorded by the TNHIRD.

### Patient Selection

**Figure 1:**
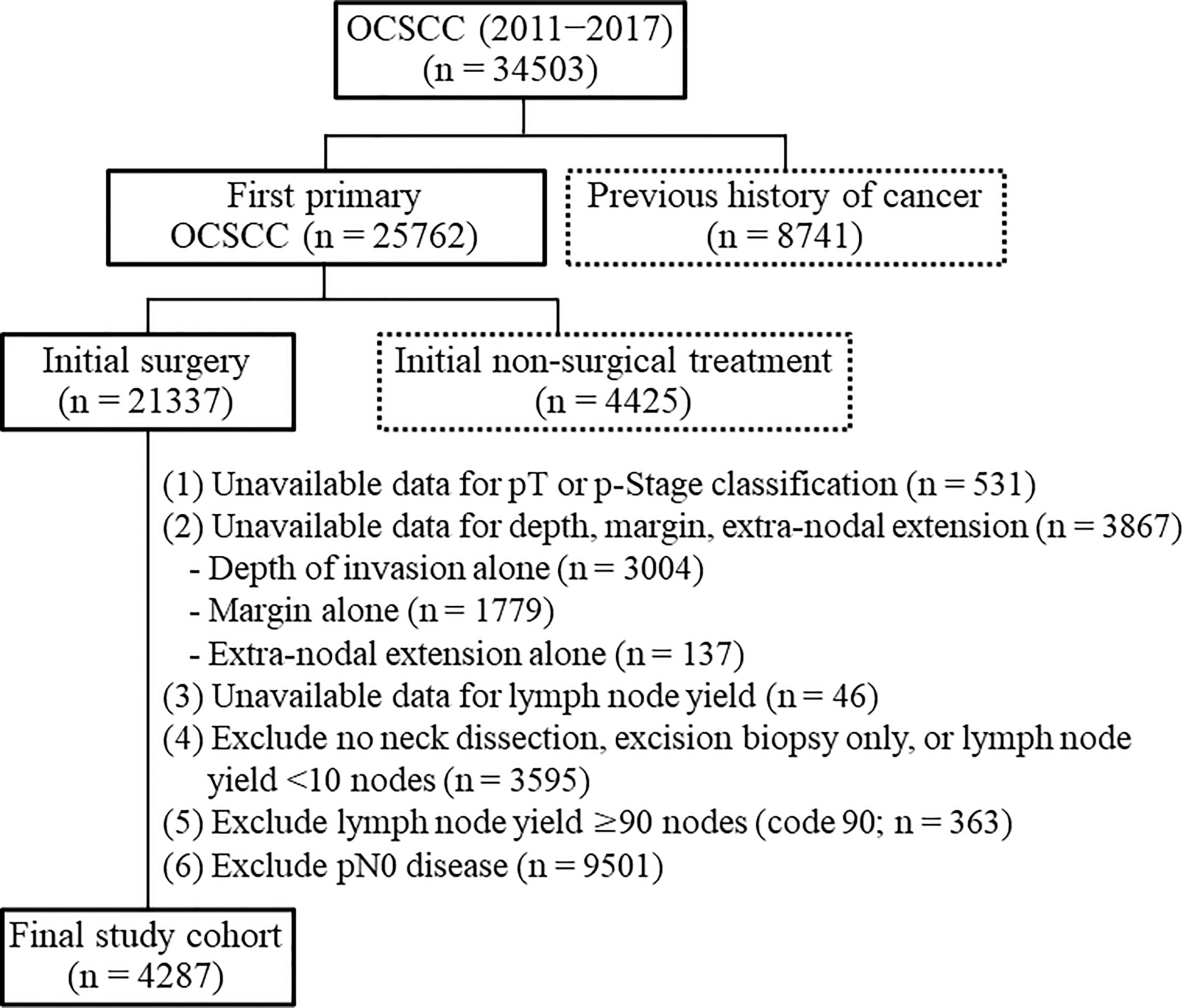
Flow of patients through the study.

Patients diagnosed with OCSCC between 2011 and 2017 were eligible for inclusion. Cases were selected according to the following International Classification of Diseases for Oncology, Third Edition [ICDO-3] codes: lip cancer [C00.0; C00.1; C00.2; C00.3; C00.4; C00.5; C00.6; C00.8; C00.9], tongue cancer [C02.0; C02.1; C02.2; C02.3; C02.8; C02.9], alveolar ridge cancer [C03.0; C03.1; C03.9], floor of mouth cancer [C04.0; C04.1; C04.8; C04.9], hard palate cancer [C05.0; C05.8; C05.9], buccal cancer [C06.0], retromolar trigone cancer [C06.2], and other forms of oral cavity cancer [C06.1; C06.8; C06.9]). We followed-up the study participants until December 2019. A study flowchart is shown in **Figure 1**. Exclusion criteria were as follows: previous history of cancer (n = 8741), initial treatment different from surgery (n = 4425), unknown pathological stage (n = 531), unavailable data for depth, margins, and ENE (n = 3867), unavailable data for lymph node yield (n = 46), no neck dissection, excision biopsy only, or lymph node yield <10 nodes (n = 3595), lymph node yield ≥90 nodes (n = 363), and pN0 disease (n = 9501). Patients with a nodal yield of less than 10 nodes were excluded because of an incomplete neck node dissection. We also excluded cases with a nodal yield ≥ 90 (code 90) because the actual number of harvested nodes was unknown. The final study cohort consisted of 4287 patients.

### Statistical Analysis

The cut-off points for LNR, the number of pathologically positive nodes, and the log odds of positive lymph were the break points in the log hazard function; they were identified by examining the functional relationships between the three variables and the hazard ratios for DSS and OS. To this aim, we built a Cox proportional hazards model with a spline function in the R statistical environment. The log odds ratio was calculated with the following formula:


$$\text{log}\left\{\frac{\text{positive lymph nodes}+0.5}{\text{lymph node ratio}-\left(\text{positive lymph nodes}+0.5\right)}\right\}$$

The primary survival endpoints were the 5-year DSS and OS rates. The duration of follow-up was calculated from the date of surgery to the date of death. Patients who were alive at the time of last follow-up were right-censored. Survival curves were plotted with the Kaplan-Meier method and compared using the log-rank test. Cox proportional hazards regression analysis was applied to estimate hazard ratios (HRs) and 95% confidence intervals (CIs) for survival endpoints after allowance for potential confounders. The multivariable model was adjusted using all variables entered in univariable analysis by applying a stepwise selection procedure. All calculations were performed with SAS (version 9.4) and R (version 4.0.2). A two-tailed *p* value of 0.05 or less was considered statistically significant.

## Results

### Optimal Cut-Off Values for Nodal Parameters

**Figure 2:**
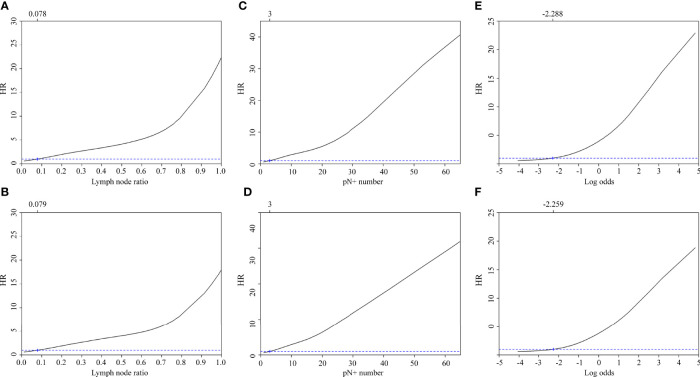
Adjusted hazard ratios for 5-year disease-specific survival and overall survival according to the lymph node ratio **(A, B)**, the number of pathologically positive nodes **(C, D)**, and the log odds of positive lymph nodes **(E, F)**.

Cox proportional hazards regression analysis identified the following optimal cut-off values for LNR, number of pathologically positive nodes, and log odds of positive lymph nodes: 0.078 (5-year DSS) and 0.079 (5-year OS); three pathologically positive nodes (5-year DSS) and three pathologically positive nodes (5-year OS); -2.288 (5-year DSS) and -2.259 (5-year OS), respectively (**Figures 2A–F**).

### Patient Characteristics According to the Lymph Node Ratio

Compared with patients with a LNR <0.078/0.079 (**Table 1**), those with a LNR ≥0.078/0.079 had a significantly higher prevalence of the following parameters: female sex, age ≥65 years, pT4, pN3b, pStage IV, depth ≥10 mm, margin status <5 mm, ENE, adjuvant therapy, presence of at least three pathologically positive nodes, and log odds of positive lymph nodes *≥*-2.288/-2.259. There was a marked overlap (nearly 98%) between cases with a LNR ≥0.078/0.079 and those with a log odds of positive lymph nodes ≥-2.288/-2.259.

**Table 1:** General characteristics of patients with oral cavity squamous cell carcinoma stratified according to the lymph node ratio (cut-off value for 5-year disease-specific survival: 0.078; cut-off value for 5-year overall survival: 0.079).

Characteristic (n, %)	LNR <0.078 (n = 2689)	LNR ≥0.078 (n = 1598)	*p*	LNR <0.079 (n = 2706)	LNR ≥0.079 (n = 1581)	*p*
Sex			0.0025			0.0013
Male (3857, 90.0)	2448 (91.0)	1409 (88.2)		2465 (91.1)	1392 (88.1)	
Female (430, 10.0)	241 (9.0)	189 (11.8)		241 (8.9)	189 (11.9)	
Age (years)			0.0242			0.0210
<65 (3647, 85.1)	2313 (86.0)	1334 (83.5)		2328 (86.0)	1319 (83.4)	
≥65 (640, 14.9)	376 (14.0)	264 (16.5)		378 (14.0)	262 (16.6)	
Pathologic T status			<0.0001			<0.0001
T1 (336, 7.8)	248 (9.2)	88 (5.5)		249 (9.2)	87 (5.5)	
T2 (1215, 28.3)	826 (30.7)	389 (24.3)		827 (30.6)	388 (24.5)	
T3 (830, 19.4)	532 (19.8)	298 (18.7)		537 (19.8)	293 (18.5)	
T4 (1906, 44.5)	1083 (40.3)	823 (51.5)		1093 (40.4)	813 (51.5)	
Pathologic N status			<0.0001			<0.0001
pN1 (1242, 29.0)	1196 (44.5)	46 (2.9)		1196 (44.2)	46 (2.9)	
pN2 (1423, 33.2)	903 (33.6)	520 (32.5)		911 (33.7)	512 (32.4)	
pN3a (3, 0.1)	3 (0.1)	0 (0.0)		3 (0.1)	0 (0.0)	
pN3b (1619, 37.8)	587 (21.8)	1032 (64.6)		596 (22.0)	1023 (64.7)	
Pathologic stage			<0.0001			<0.0001
III (838, 19.5)	801 (29.8)	37 (2.3)		801 (29.6)	37 (2.4)	
IV (3449, 80.5)	1888 (70.2)	1561 (97.7)		1905 (70.4)	1544 (97.6)	
Depth of invasion			<0.0001			<0.0001
<10 mm (1553, 36.2)	1072 (39.9)	481 (30.1)		1074 (39.7)	479 (30.3)	
≥10 mm (2734, 63.8)	1617 (60.1)	1117 (69.9)		1632 (60.3)	1102 (69.7)	
Margin status			<0.0001			<0.0001
<5 mm (2399, 56.0)	1428 (53.1)	971 (60.8)		1434 (53.0)	965 (61.0)	
≥5 mm (1888, 44.0)	1261 (46.9)	627 (39.2)		1272 (47.0)	616 (39.0)	
Extra-nodal extension			<0.0001			<0.0001
Absent (2283, 53.3)	1737 (64.6)	546 (34.2)		1745 (64.5)	538 (34.0)	
Present (2004, 46.7)	952 (35.4)	1052 (65.8)		961 (35.5)	1043 (66.0)	
Treatment modality			<0.0001			<0.0001
S alone (632, 14.7)	444 (16.5)	188 (11.8)		446 (16.5)	186 (11.8)	
S plus CT or S plus RT or S plus CT and RT (3655, 85.3)	2245 (83.5)	1410 (88.2)		2260 (83.5)	1395 (88.2)	
Number of pathologically positive nodes			<0.0001			<0.0001
<3 (2665, 62.2)	2316 (86.1)	349 (21.8)		2316 (85.6)	349 (22.1)	
≥3 (1622, 37.8)	373 (13.9)	1249 (78.2)		390 (14.4)	1232 (77.9)	
Log odds – OS						<0.0001
-2.259 (2538, 59.2)				2587 (95.6)	33 (2.1)	
≥ -2.259 (1749, 40.8)				119 (4.4)	1548 (97.9)	
Log odds – DSS			<0.0001			
< -2.288 (2620, 61.1)	2503 (93.1)	35 (2.2)				
≥ -2.288 (1667, 38.9)	186 (6.9)	1563 (97.8)				

LNR, lymph node ratio; S, surgery; CT, chemotherapy; RT, radiotherapy; OS, overall survival; DSS, disease-specific survival.

### Patient Characteristics According to the Log Odds of Positive Lymph Nodes

Compared with patients with a log odds of positive lymph nodes <-2.288/-2.259 (**Supplementary Table 1**), those with a log odds ≥-2.288/-2.259 had a significantly higher prevalence of the following variables: female sex, age ≥65 years, pT4, pN3b, pStage IV, depth ≥10 mm, margin status <5 mm, ENE, adjuvant therapy, and presence of at least three pathologically positive nodes.

### Five-Year Survival Rates

In the entire study cohort (n = 4287), the 5-year DSS and OS rates were 57% and 48%, respectively. The 5-year survival rates of patients with a LNR <0.078 *versus* ≥0.078 (DSS) and <0.079 *versus* ≥0.079 (OS) were 65%/44% (*p <*0.0001) and 56%*/*34% (*p <*0.0001), respectively. The 5-year survival rates of patients with less than three pathologically positive nodes *versus* at least three pathologically positive nodes were 66%/43% (DSS; *p <*0.0001) and 57%*/*32% (OS; *p <*0.0001), respectively

**Figure 3:**
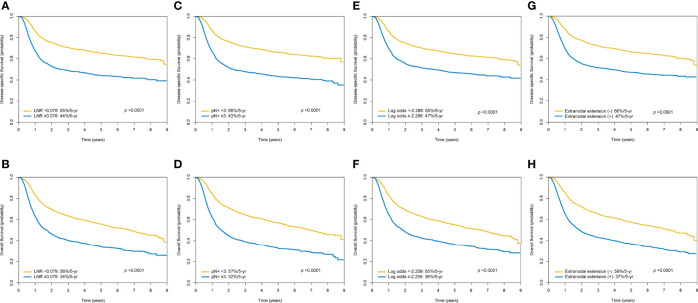
Kaplan-Meier plots of 5-year disease-specific survival and overall survival according to the lymph node ratio **(A, B)**, the number of pathologically positive nodes **(C, D)**, the log odds of positive lymph nodes **(E, F)**, and the presence of extra-nodal extension **(G, H)**.

The 5-year survival rates of cases with a log odds of positive lymph nodes <-2.288 *versus* ≥-2.288 (DSS) and <-2.259 *versus* ≥-2.259 (OS) were 65%/47% (*p <*0.0001) and 55%*/*36% *p <*0.0001), respectively. The 5-year survival rates of patients with absence of ENE *versus* presence of ENE were 66%/47% (DSS; *p <*0.0001) and 59%*/*37% (OS; *p <*0.0001), respectively (**Figures 3A–H**).

### Univariable and Multivariable Analyses

**Table 2** shows the univariable and multivariable associations between the study variables and the 5-year DSS and OS rates. After adjustment for potential confounders in multivariable analyses, we identified three nodal-related variables (LNR ≥0.078/0.079, the presence of at least three pathologically positive nodes, and ENE) as independent RFs for both 5-year DSS and OS rates.

**Table 2:** Univariable and multivariable analyses of risk factors for 5-year disease-specific survival and overall survival in the entire study cohort (n = 4287).

Risk factor	Disease-specific survival				Overall survival			
	Univariable analysis		Multivariable analysis		Univariable analysis		Multivariable analysis	
	HR (95% CI)	*p*	HR (95% CI)	*p*	HR (95% CI)	*p*	HR (95% CI)	*p*
Lymph node ratio								
<0.078/<0.079	1		1		1		1	
≥0.078/≥0.079	2.02 (1.84-2.22)	<0.0001	1.41 (1.25-1.59)	<0.0001	1.94 (1.79-2.11)	<0.0001	1.41 (1.27-1.58)	<0.0001
Log odds								
< -2.288/-2.258	1		–		1		1	
≥ -2.288/≥-2.258	1.83 (1.67-2.01)	<0.0001	–	ns	1.79 (1.65-1.94)	<0.0001		ns
Number of pathologically positive nodes								
< 3	1		1		1		1	
≥ 3	2.16 (1.97-2.37)	<0.0001	1.33 (1.17-1.51)	<0.0001	2.10 (1.93-2.28)	<0.0001	1.35 (1.20-1.51)	<0.0001
Extra-nodal extension								
Absent	1		1		1		1	
Present	1.95 (1.78-2.15)	<0.0001	1.44 (1.30-1.60)	<0.0001	1.92 (1.77-2.09)	<0.0001	1.53 (1.40-1.68)	<0.0001
Sex								
Male	1.02 (0.87-1.19)	0.8270	–	ns	1.10 (0.95-1.27)	0.2076	1.21 (1.04-1.39)	0.0120
Female	1		–		1		1	
Age (years)								
<65	1		1		1		1	
≥65	1.28 (1.14-1.45)	<0.0001	1.22 (1.08-1.39)	0.0015	1.45 (1.30-1.62)	<0.0001	1.42 (1.27-1.58)	<0.0001
Pathologic T status								
T1	1		1		1		1	
T2	1.48 (1.17-1.88)	0.0013	1.43 (1.13-1.82)	0.0034	1.44 (1.17-1.77)	0.0006	1.43 (1.17-1.77)	0.0006
T3	1.95 (1.53-2.48)	<0.0001	1.74 (1.36-2.22)	<0.0001	1.96 (1.59-2.42)	<0.0001	1.88 (1.52-2.32)	<0.0001
T4	2.86 (2.28-3.58)	<0.0001	2.28 (1.80-2.88)	<0.0001	2.74 (2.26-3.33)	<0.0001	2.46 (2.02-3.00)	<0.0001
Pathologic N status								
pN1	1		–		1		–	
pN2	1.62 (1.42-1.86)	<0.0001	–	ns	1.53 (1.36-1.72)	<0.0001	–	ns
pN3a	1.50 (0.21-10.65)	0.6875	–	ns	1.09 (0.15-7.76)	0.9309	–	ns
pN3b	2.79 (2.46-3.16)	<0.0001	–	ns	2.62 (2.36-2.93)	<0.0001	–	ns
Pathologic stage								
III	1		1		1		–	
IV	2.61 (2.25-3.04)	<0.0001	1.25 (1.04-1.51)	0.0174	2.26 (1.99-2.56)	<0.0001	–	ns
Depth of invasion								
<10 mm	1		–		1		–	
≥10 mm	1.70 (1.53-1.88)	<0.0001	–	ns	1.68 (1.53-1.84)	<0.0001	–	ns
Margin status								
<5 mm	1.19 (1.09-1.31)	0.0002	1.12 (1.02-1.23)	0.0201	1.22 (1.12-1.33)	<0.0001	1.17 (1.08-1.28)	0.0002
≥5 mm	1		1		1		1	
Treatment modality								
S alone	1.09 (0.96-1.24)	0.1765	1.38 (1.21-1.57)	<0.0001	1.189(1.06-1.33)	0.0028	1.53 (1.36-1.72)	<0.0001
S plus CT or S plus RT or S plus CT and RT	1		1		1		1	

HR, hazard ratio; CI, confidence interval; ns, not significant; S, surgery; CT, chemotherapy; RT, radiotherapy.

### Prognostic Scoring System

We therefore devised a four-point prognostic scoring system (range: 0−3) according to the presence or absence of each nodal parameter identified as an independent prognostic RFs, as follows: 0 for a LNR <0.078/0.079 and 1 for a LNR ≥0.078/0.079; 0 for less than three pathologically positive nodes and 1 for at least three pathologically positive nodes; 0 for the absence of ENE and 1 for the presence of ENE. Based on the prognostic score, we identified the following four risk groups for 5-year DSS and OS: score 0, score 1, score 2, and 3.

The number of patients with a score of 0, 1, 2, and 3 was 1611/1048/708/920 (DSS) and 1611/1056/709/911 (OS), respectively.

### Five-Year Survival Rates According to the Traditional AJCC pN Classification Versus the Devised Scoring System

**Figure 4:**
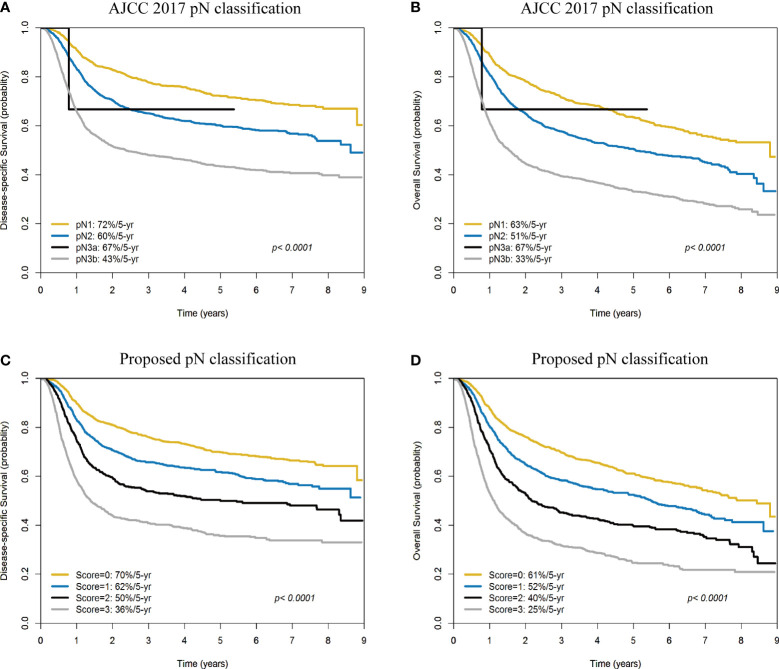
Kaplan-Meier plots of 5-year disease-specific survival **(A)** and overall survival **(B)** according to the AJCC pN classification (pN1, pN2, pN3a, and pN3b). Kaplan-Meier plots of 5-year disease-specific survival **(C)** and overall survival **(D)** according to the scoring system devised in our study (scores: 0, 1, 2, and 3).

The 5-year DSS and OS rates of the patients with pN1, pN2, pN3a, and pN3b disease were 72%/60%/67%/43% (**Figure 4A**) and 63%/51%/67%/33% (**Figure 4B**), respectively (all *p <*0.0001). The 5-year DSS and OS rates of patients with a score of 0, 1, 2, 3 according to our prognostic scoring system were 70%/62%/50%/36% (**Figure 4C**) and 61%/52%/40%/25% (**Figure 4D**), respectively (all *p <*0.0001). On applying the AJCC classification system, patients with pN3a disease showed better survival rates than those with pN2. Although the *p* values for the two classification systems (traditional AJCC *versus* scoring system proposed in our study) were similar (both <0.0001), our prognostic score showed a higher discrimination ability.

### Five-Year Disease-Specific Survival and Overall Survival in Patients With pN3b Disease According to the Prognostic Scoring System

**Figure 5:**
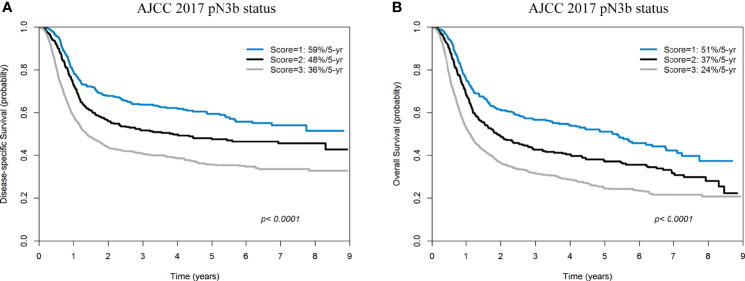
Kaplan-Meier plots of 5-year disease-specific survival **(A)** and overall survival **(B)** in patients with pN3b disease (AJCC pN classification) stratified according to a score of 1−3.

In the subgroup of patients with pN3b disease (n = 1619), 342 (21.1%), 359 (22.2%), and 918 (56.7%) cases had a score of 1, 2, and 3 (according to DSS), respectively. The 5-year DSS rates in the three groups were 59%, 48%, and 36% respectively (**Figure 5A**). In the subgroup of patients with pN3b disease, 342 (21.1%), 368 (22.7%), and 909 (56.2%) cases had a score of 1, 2, and 3 (according to OS), respectively. The 5-year OS rates in the three groups were 51%, 37%, and 24% respectively (**Figure 5B**).

## Discussion

In this nationwide registry-based study, we sought to further refine the prognostic stratification of lymph node status in patients who had undergone surgical excision of OCSCC with curative intent. To this aim, several nodal parameters – including LNR, the number of pathologically positive nodes, the log odds of positive lymph nodes, and the presence of ENE – were simultaneously taken into account. On multivariable analysis, a LNR ≥0.078/0.079, the presence of at least three pathologically positive nodes, and ENE were identified as independent predictors of survival endpoints. Interestingly, a scoring system based on these three nodal parameters outperformed the traditional AJCC pN classification in terms of survival prediction. Of note, AJCC pN classification was not retained in the multivariable model as an independent prognosticator of survival endpoints. This is likely the result of the opposite prognostic trajectories observed for patients with pN2 and pN3a disease (**Table 2**).

In the era of the AJCC 2010 staging system, several studies have investigated the independent prognostic value of LNR – among which a subset also examined whether it would improve the prognostic stratification offered the AJCC pN classification (12–36). The optimal cut-off value for LNR is not consistent in the literature, possibly because of varying statistical methods, number of participants (pN+, n = 19 to 3091), and selection of endpoints (**Supplementary Table 2**). The results of these studies revealed that LNR was an independent RF for local, regional, and/or distant control (3, 13, 14, 17–19, 25, 28, 30, 35) as well as survival outcomes (disease-free survival, DSS, and/or OS) (3, 12, 13, 15–27, 29, 31–34, 36). Previous studies that focused on the log odds of positive lymph nodes yielded discrepant findings (9–11). While some investigators (sample size with pN+ data: 77−112) found that the log odds of positive lymph nodes outperformed LNR in terms of prognostic prediction (7, 9, 10), Bao et al. (11) (sample size with pN+ data: 224) reported opposite findings (**Supplementary Table 2**).

To our knowledge, only three published studies simultaneously examined three nodal parameters (i.e., LNR, number of pathologically positive nodes, and log odds of positive lymph nodes) *versus* the traditional AJCC pN classification systems [AJCC 2010 (6, 7) or AJCC 2017 (37)] (**Supplementary Table 2** and **Table 3**). Lin et al. (6) (sample size with pN+ data: 639) found that both LNR and the log odds of positive lymph nodes outperformed the number of pathologically positive nodes in the prediction of clinical outcomes. However, Safi et al. (7) (sample size with pN+ data: 157) showed that the log odds of positive lymph nodes were prognostically superior to both LNR and the number of pathologically positive nodes. Finally, Subramaniam et al. (37) (sample size with pN+ data: 271) reported that LNR and the number of pathologically positive nodes predicted outcomes better than the log odds of positive lymph nodes. The results of our study were in accordance with those of Subramaniam and coworkers; however, on analyzing the variables associated with survival endpoints, we were able to comprehensively include a number of different RFs, which were entered in a multivariable-adjusted analysis. Conversely, the final multivariable model constructed by Subramaniam et al. (37) included only four nodal parameters (i.e., AJCC pN classification, number of pathologically positive nodes, LNR, and log odds of positive lymph nodes) and the prognostic significance of ENE was not examined. Another issue inherent in that study was that the analyses performed according to the pN2 subclassification (i.e., pN2a, pN2b, and pN2c) yielded heterogenous outcomes. In the current investigation, the 5-year DSS rates did not differ significantly between patients with pN2a (64%), pN2b (61%), and pN2c (49%) disease (*p* = 0.1181). Consequently, the three subgroups were combined into a single pN2 group for the purpose of analysis. This finding is consistent with our previous study that showed no differences in 5-year DFS rates in the three pN2 subgroups (5).

**Table 3:** Published literature focusing on the prognostic significance of lymph node ratio, log odds of positive lymph nodes, and/or number of pathologically positive nodes according to the AJCC 2017 staging system (eighth edition).

Author (years of recruitment)	AJCC staging manual (edition)	Number of patients with pathologically positive nodes (total)	Cut-offmethod	Cut-off value			Independent risk factors in multivariable analyses				
				Lymph node ratio	Log oddsof positive lymph nodes	Number of pathologically positive nodes	NC	DM	DFS	DSS	OS
Liao CT (2011–2017) current study	Eighth	4287 (13027)	Hazard ratios	0.078 and 0.079	-2.288 and -2.259	1-2/≥3	–	–	–	**√**	**√**
^38^Subramaniam N (2004-2014)	Eighth	271 (643)	Derived from the published literature	0/0.1/0.4	-1.69/-1.29/ -0.88	0/1-2/ 3-4/≥5	–	–	**√**	–	**√**
^39^Rajappa SK (2009-2017)	Eighth	466 (1431)	–	–	–	0/1/2/≥3	–	–	**√**	–	**√**
^5^Liao CT (1996-2017)	Eighth	365-pN3b	Kaplan-Meier	–	–	<8/≥8	**√**	**√**	–	**√**	**√**
^40^Agarwal JP (2011)	Eighth	94 (120)	Log-rank test	0.12	–	–	–	–	**√**	–	–

AJCC, American Joint Committee on Cancer; NC, neck control; DM, distant metastasis; DFS, disease-free survival; DSS, disease-specific survival; OS, overall survival.

Herein, the outcomes of patients with pN3a were more favorable than those of cases with pN2 disease. Notably, pN3a disease according to the AJCC Staging Manual, eighth edition (i.e., presence of a single metastatic node >6 cm in size without evidence of ENE – a condition which was previously staged as pN3 disease according to the AJCC Staging Manual seventh edition) is an extremely uncommon condition (<0.1% of all OCSCC cases, 0% [0/1788] in AJCC 2017 Staging Manual, 0% [0/1933] in Liao et al.’s study, 0.08% [6/6887] in the Surveillance, Epidemiology, and End-Results [SEER] database) (4, 5, 42). In the current study, pN3a disease was identified in 0.07% of the study patients (3/4287) and the question as to whether their outcomes were different from those with pN2 disease remains unanswered. Interestingly, the prognostic scoring system devised in our study resulted in an effective stratification of patients with pN3b disease (**Figures 5A, B**). A larger sample size of patients with pN3a disease could have improved the statistical power of the study in terms of identifying significant intergroup differences.

According to the AJCC 2017 Staging Manual (eighth edition), the pathological node classification is based on the number of positive nodes (single *versus* multiple) and the presence of ENE. Subramaniam et al. (37) have previously proposed a categorical classification based on the number of pathologically positive nodes (i.e., 0 *versus* 1−2 *versus* 3−4 *versus* ≥5 nodes) as a simple and clinically convenient strategy to replace the traditional AJCC pN staging system. Similarly, Ho et al. (42) have proposed a categorical classification based on the number of pathologically positive nodes and ENE (i.e., 0 LN+ *versus* 1 LN+/ENE[-] *versus* 2 LN+ or 1 LN+/ENE[+] *versus* 3-7 LN+ *versus* ≥8 LN+). It can be argued that the scoring system devised in our study may be less intuitive for clinical application in a real-world setting. Even so, for patients with OCSCC and nodal spread, we identified other nodal parameters (i.e., LNR and ENE) that had an independent effect on survival outcomes. Further validation of the proposed nodal classification scheme is necessary before widespread clinical implementation.

This study has some important limitations. First, the nationwide registry that served as data source for this study did not contain information on local, regional, and distant events. Therefore, our analysis was limited to 5-year DSS and OS. Second, data concerning certain pathological RFs (e.g., perineural invasion and lymphovascular invasion) were included in the TCRD as of 2018 only; for that reason, their associations with survival outcomes should be investigated in future studies. Finally, the registry-based nature of the study may be associated with information bias. It is also possible that the geographic setting in which the study was conducted (i.e., a betel quid chewing endemic area) could have limited the generalizability of the findings and, for that reason, additional research in Western countries is required.

## Conclusions

In this nationwide study comprising a large sample of Taiwanese patients with resected OCSCC, three nodal parameters (i.e., a LNR ≥0.078/0.079, the presence of at least three pathologically positive nodes, and ENE) assessed in combination provided a better prognostic stratification than the traditional AJCC pN classification.

## Data Availability Statement

The original contributions presented in the study are included in the article/**Supplementary Material**. Further inquiries can be directed to the corresponding author.

## Ethics Statement

The study was approved by the Chung Gung Memorial Hospital Institutional Review Board (IRB; approval number: 201801398B0A3). The requirement for written informed consent was waived due to the retrospective study design.

## Author Contributions

Conception and design: C-JK, Y-WW, and C-TL. Analysis and interpretation of data: All authors. Drafting the article or revising it critically for important intellectual content: All authors. Final approval of manuscript: All authors. Agreement to be accountable for all aspects of the work: All authors.

## Funding

This research was financially supported by grants (CMRPD1H0521 and BMRPC55) from the Chang Gung Medical Research Program.

## Conflict of Interest

The authors declare that the research was conducted in the absence of any commercial or financial relationships that could be construed as a potential conflict of interest.

## Publisher’s Note

All claims expressed in this article are solely those of the authors and do not necessarily represent those of their affiliated organizations, or those of the publisher, the editors and the reviewers. Any product that may be evaluated in this article, or claim that may be made by its manufacturer, is not guaranteed or endorsed by the publisher.
