# Supplementary material for: Towards an Improved Pathological Node Classification for Prognostic Stratification of Patients With Oral Cavity Squamous Cell Carcinoma: Results From a Nationwide Registry Study
Source: Front Oncol. 2022 Jun 28;12:910158. doi: 10.3389/fonc.2022.910158 (PMC9273780; doi:10.3389/fonc.2022.910158)
Supplement: Supplementary file 1 [file Table_1.doc]

**Supplementary Table 1.** General characteristics of patients with oral cavity squamous cell carcinoma stratified according to the log odds of positive lymph nodes (cut-off value for 5-year disease-specific survival: -2.288; cut-off value for 5-year overall survival: -2.259)

| Characteristic | | Log odds  <-2.288  (n = 2538) | Log odds  ≥-2.288  (n = 1749) | *p* | Log odds  <-2.259  (n = 2620) | Log odds  ≥-2.259  (n = 1667) | *p* |
| --- | --- | --- | --- | --- | --- | --- | --- |
| Sex | |  |  | 0.0008 |  |  | 0.0004 |
|  | Male (3857, 90.0) | 2316 (91.3) | 1541 (88.1) |  | 2391 (91.3) | 1466 (87.9) |  |
|  | Female (430, 10.0) | 222 (8.7) | 208 (11.9) |  | 229 (8.7) | 201 (12.1) |  |
| Age (years) | |  |  | 0.0013 |  |  | 0.0006 |
|  | <65 (3647, 85.1) | 2196 (86.5) | 1451 (83.0) |  | 2268 (86.6) | 1379 (82.7) |  |
|  | ≥65 (640, 14.9) | 342 (13.5) | 298 (17.0) |  | 352 (13.4) | 288 (17.3) |  |
| Pathologic T status | |  |  | <0.0001 |  |  | <0.0001 |
|  | T1 (336, 7.8) | 224 (8.8) | 112 (6.4) |  | 233 (8.9) | 103 (6.2) |  |
|  | T2 (1215, 28.3) | 756 (29.8) | 459 (26.2) |  | 783 (29.9) | 432 (25.9) |  |
|  | T3 (830, 19.4) | 504 (19.9) | 326 (18.6) |  | 517 (19.7) | 313 (18.8) |  |
|  | T4 (1906, 44.5) | 1054 (41.5) | 852 (48.8) |  | 1087 (41.5) | 819 (49.1) |  |
| Pathologic N status | |  |  | <0.0001 |  |  | <0.0001 |
|  | pN1 (1242, 29.0) | 1103 (43.5) | 139 (8.0) |  | 1128 (43.1) | 114 (6.8) |  |
|  | pN2 (1423, 33.2) | 852 (33.6) | 571 (32.7) |  | 884 (33.7) | 539 (32.3) |  |
|  | pN3a (3, 0.1) | 3 (0.1) | 0 (0.0) |  | 3 (0.1) | 0 (0.0) |  |
|  | pN3b (1619, 37.8) | 580 (22.9) | 1039 (59.4) |  | 605 (23.1) | 1014 (60.8) |  |
| Pathologic stage | |  |  | <0.0001 |  |  | <0.0001 |
|  | III (838, 19.5) | 730 (28.8) | 108 (6.2) |  | 748 (28.6) | 90 (5.4) |  |
|  | IV (3449, 80.5) | 1808 (71.2) | 1641 (93.8) |  | 1872 (71.4) | 1577 (94.6) |  |
| Depth of invasion | |  |  | <0.0001 |  |  | <0.0001 |
|  | <10 mm (1553, 36.2) | 989 (39.0) | 564 (32.3) |  | 1021 (39.0) | 532 (31.9) |  |
|  | ≥10 mm (2734, 63.8) | 1549 (61.0) | 1185 (67.7) |  | 1599 (61.0) | 1135 (68.1) |  |
| Margin status | |  |  | <0.0001 |  |  | <0.0001 |
|  | <5 mm (2399, 56.0) | 1333 (52.5) | 1066 (61.0) |  | 1387 (52.9) | 1012 (60.7) |  |
|  | ≥5 mm (1888, 44.0) | 1205 (47.5) | 683 (39.0) |  | 1233 (47.1) | 655 (39.3) |  |
| Extra-nodal extension | |  |  | <0.0001 |  |  | <0.0001 |
|  | Absent (2283, 53.3) | 1618 (63.8) | 665 (38.0) |  | 1667 (63.6) | 616 (37.0) |  |
|  | Present (2004, 46.7) | 920 (36.2) | 1084 (62.0) |  | 953 (36.4) | 1051 (63.0) |  |
| Treatment modality | |  |  | <0.0001 |  |  | 0.0003 |
|  | S alone (632, 14.7) | 421 (16.6) | 211 (12.1) |  | 427 (16.3) | 205 (12.3) |  |
|  | S plus CT or S plus RT or S plus CT and RT  (3655, 85.3) | 2117 (83.4) | 1538 (87.9) |  | 2193 (83.7) | 1462 (87.7) |  |
| Number of pathologically positive nodes | |  |  | <0.0001 |  |  | <0.0001 |
|  | <3 (2665, 62.2) | 2130 (83.9) | 535 (30.6) |  | 2197 (83.9) | 468 (28.1) |  |
|  | ≥3 (1622, 37.8) | 408 (16.1) | 1214 (69.4) |  | 423 (16.1) | 1199 (71.9) |  |

*Abbreviation:* S, surgery; CT, chemotherapy; RT, radiotherapy.
