# Supplementary material for: Towards an Improved Pathological Node Classification for Prognostic Stratification of Patients With Oral Cavity Squamous Cell Carcinoma: Results From a Nationwide Registry Study
Source: Front Oncol. 2022 Jun 28;12:910158. doi: 10.3389/fonc.2022.910158 (PMC9273780; doi:10.3389/fonc.2022.910158)
Supplement: Supplementary file 2 [file Table_2.doc]

| **Supplementary Table 2.** Published literature focusing on the prognostic significance of lymph node ratio, log odds of positive lymph nodes, and/or number of pathologically positive nodes prior to the introduction of the AJCC 2017 staging system (eighth edition) for oral cavity cancer | | | | | | | | | | | | | |
| --- | --- | --- | --- | --- | --- | --- | --- | --- | --- | --- | --- | --- | --- |
| Author (years of recruitment) | AJCC staging manual (edition) | Number of patients with pathologically positive nodes (total) | Cut-off method | Cut-off value | | | Independent risk factors in multivariable analyses | | | | | | |
|  |  |  |  | Lymph node  ratio | Log odds  of positive lymph nodes | Number of pathologically positive nodes | LC | NC | LRC | DM | DFS | DSS | OS |
| ^6^Lin NC (2008-2018) | Seventh | 639 | Hazard ratio | 0.05/0.129 | -1.5/-1/-0.5 | 0/1/2/3-4/5 | - | - | - | - | √ | - | - |
| ^7^Safi AF (2002-2013) | Seventh | 157 (499) | ROC | 0.07 | -0.968 | 0-5/>5 | - | - | √ | - | - | - | - |
| ^8^Lee CC (SEER, 2007-2013) | Seventh | 1826 (3958) | Hazard ratio | 0/0.2/0.4 | -1.68/-1.29/-0.88 |  | - | - | - | - | - | √ | √ |
| ^9^Lee CC (2004-2013) | Seventh | 112 (347) | Hazard ratio | 0/0.2/0.4 | -1.58/-1.26/  -0.82 |  | - | - | - | - | - | - | √ |
| ^10^Jin W (2009-2013) | NM | 77 (233) | X-tile | 0.024/0.133 | -1.491/-0.763 |  | - | - | - | - | - | - | √ |
| ^11^Bao X (2005-2017) | NM | 244 (706) | X-lite |  | 0.00/0.01 | 0/1-2/3-40 | - | - | - | - | - | - | √ |
| ^12^Lee H (2006-2015) | Seventh | 149 (345) | ROC | 0.05/0.1 |  | 0/1/2-4/≥5 | - | - | - | - | √ | √ | √ |
| ^13^Liao CT (1996-2004) | Fifth | 318 (889) |  |  |  | <5/≥5 | - | - | - | √ | - | - | - |
| ^14^Kang CJ (1996-2008) | Fifth | 72 (102) |  |  |  | <4/≥4 |  | √ | - | √ | √ | √ | √ |
| ^15^Urban D (SEER, 1988-2007) | Sixth | 3091 | Maximally selected rank | 0.06/0.125 |  |  | - | - | - | - | -- |  | √ |
| ^16^Patel SG (1990-2011) | Seventh | 1986 (4254) | ROC | 0.07 |  |  | - | - | - | - | - | √ | √ |
| ^17^Sayed (2001-2010) | Sixth | 725 (1408) | ROC | 0.088 |  |  | - | √ | - | - | √ | - | √ |
| ^3^Liao CT (1996-2009) | Seventh | 309 (I-III)  148 (I-V) | Kaplan-Meier | 0.16 (I-III)  0.048 (I-V) |  |  | -  √ | √  - | -  - | √  - | √  - | √  - | -  √ |
| ^18^Hosni A (1994-2012) | Seventh | 432 (914) | Median | 0.06 |  |  |  | √ | - | √ | - | - | √ |
| ^19^Gil Z (1986-1996) | NM | 167 (386) | Median | 0.06 |  |  | - | - | √ | - | - | √ | - |
| ^20^Ebrahimi A (1987-2009) | Seventh | 165 (313) | Log-transformation | 0.025/0.075/ 0.2 |  |  | - | - | - | - | - | √ | √ |
| ^21^Lee H (2006-2015) | Seventh | 149 (345) | ROC | 0/0.05/0.1 |  |  | - | - | - | - | √ | √ | √ |
| ^22^Kunzel J (1980-2010) | Seventh | 148 (374) | ROC | 0.05 |  |  | - | - | - | - | - | √ | - |
| ^23^Shrime MG (1994-2004) | NM | 143 | Maximally selected rank | 0.06/0.13 |  |  | - | - | - | - | - | - | √ |
| ^24^Chang WC (2002-2015) | Seventh | 133 (389) | ROC | 0.05 |  |  | - | - | - | - | √ | - | √ |
| ^25^Adel M (2008-2013) | Sixth | 125 (277) | Kaplan-Meier | 0.06 |  |  | - | - | - | - | √ | - | √ |
| ^26^Ding (2000-2015) | Seventh | 86 (149) | Median | 0.1 |  |  | - | - | - | √ | √ | - | √ |
| ^27^Lieng H (1980-2011) | Seventh | 72 | Minimum p-value | 0.143 |  |  | - | - | - | - | √ | - | √ |
| ^28^Son HJ (2010-2015) | Seventh | 65 (157) | ROC | 0.05 |  |  | - | - | - | - | √ | √ | √ |
| ^29^Safi AF (2003-2013) | Seventh | 49 (130) | Median | 0.06 |  |  | - | - | √ | - | - | - | - |
| ^30^Zirk M (2002-2013) | Seventh | 47 (155) | NM | 0.07 |  |  | - | - | - | - | - | - | √ |
| ^31^Safi AF (2002-2013) | Seventh | 45 (89) | Median | 0.07 |  |  | - | - | √ | - | - | - | - |
| ^32^Ong W (2002-2010) | Seventh | 42 (99) | Derived from the published literature | 0.06 |  |  | - | - | - | - | - | √ | √ |
| ^33^Yamagata K (2008-2015) | Seventh | 36 (95) | ROC | 0.04 |  |  | - | - | - | - | - | - | √ |
| ^34^Suzuki H (2008-2013) | Seventh | 35 | Log-rank test | 0.07 |  |  | - | - | - | √ | - | - | √ |
| ^35^Kim KY (1994-2003) | Sixth | 31 (95) | First quartile  of the number of dissected nodes (reference) | 0.04 |  |  | - | - | - | - | - | - | √ |
| ^36^Safi AF (2003-2013) | Seventh | 30 (95) | Median | 0.07 |  |  | - | - | √ | - | - | - | - |
| ^37^Chow TL (2000-2016) | Seventh | 19 (39) | Derived from the published literature | 0.07 |  |  | - | - | - | - | - | - | √ |

Abbreviations: AJCC, American Joint Committee on Cancer; SEER, Surveillance, Epidemiology, and End-Results; LC, local control; NC, neck control; LRC, locoregional control; DM, distant metastasis; DFS, disease-free survival; DSS, disease-specific survival; OS, overall survival; NM, not mentioned; ROC, receiver operating characteristic.
